# Scout-Triggered Multiple Reaction Monitoring Enables Robust Quantification of Host Cell Proteins Across Bioprocess Matrices

**DOI:** 10.3390/proteomes14010009

**Published:** 2026-02-17

**Authors:** Julie Flecheux, Chloé Bardet, Laura Herment, Tanguy Fortin, Jérôme Lemoine

**Affiliations:** 1 Institut des Sciences Analytiques (ISA), UMR5280, Université Claude Bernard Lyon1, CNRS, 69100 Villeurbanne, France; 2 ANAQUANT, 69007 Lyon, France; chloe.bardet@anaquant.com (C.B.); laura.herment@anaquant.com (L.H.); tanguy.fortin@anaquant.com (T.F.)

**Keywords:** antibody(s) (mAbs), bioprocess development, host cell protein (HCP), liquid chromatography-mass spectrometry (LC-MS), multiple reaction monitoring (MRM), proteomic

## Abstract

Background: Host cell proteins (HCPs) are process-related impurities that must be monitored in biopharmaceutical products due to their potential impact on product quality and patient safety. Targeted LC–MS/MS approaches such as multiple reaction monitoring (MRM) enable protein-specific HCP quantification but are difficult to apply in highly multiplexed assays because of retention time (RT) variability across complex bioprocess matrices. Methods: Here, we show that conventional RT-scheduled MRM workflows lack transferability when applied to heterogeneous drug substances and process intermediates. Using a targeted assay comprising 240 peptides corresponding to 97 CHO-derived HCPs, RT shifts of several minutes resulted in truncated chromatographic peaks and peptide signal loss, even when wide scheduling windows were used. To overcome this limitation, a scout-triggered MRM (st-MRM) acquisition strategy based on event-driven monitoring was implemented. Results: This approach enabled robust peptide detection across diverse matrices within a single injection, without method re-optimization. Absolute quantification using stable isotope-labeled peptides spanned six orders of magnitude, with HCPs quantified down to 2.9 ppm in purified drug substances. Conclusion: Overall, st-MRM improves the robustness and transferability of highly multiplexed targeted proteomics workflows for HCP analysis.

## 1. Introduction

In 2022, 435 biopharmaceutical active ingredients were listed in Europe and/or the United States [1]. Among them, recombinant therapeutic proteins, particularly monoclonal antibodies (mAbs), are expressed in host cells, typically mammalian cells such as Chinese hamster ovary (CHO) cells, in nearly 70% of cases [2,3]. Host cell proteins (HCPs) are residual impurities that can remain in final biopharmaceutical products and are not entirely eliminated during filtration and chromatographic enrichment processes. HCPs pose a risk to patient safety as they can be toxic or induce an immune response [4,5]. Furthermore, some HCPs may have proteolytic activity, thereby compromising the integrity and efficacy of the therapeutic molecule. For example, the cathepsin D protease has been identified in CHO cultures as responsible for the degradation of a recombinant fusion Fc protein [6]. On the other hand, lipoprotein lipase (LPL) has been shown to be the cause of degradation of polysorbate 80 (PS-80) and polysorbate 20 (PS-20) [7], compounds commonly added to pharmaceutical products to protect monoclonal antibodies from degradation during purification, filtration, lyophilization, storage, and final delivery [8].

This is why regulatory agencies such as the Food and Drug Administration (FDA) and the European Medicines Agency (EMA) require, among the various quality control points for each bioproduction batch, the measurement of the total amount of HCP in the final pharmaceutical product, which must not exceed 100 ppm according to the standard [9,10,11].

In industrial settings, total HCP levels are most commonly monitored using enzyme-linked immunosorbent assays (ELISA), which provide sensitive and high-throughput measurements of global HCP content. However, ELISA does not provide protein-specific information and relies on antibody coverage that may vary depending on the host cell line and immunogen used [5,12,13,14].

Liquid chromatography coupled to tandem mass spectrometry (LC–MS/MS) has therefore emerged as a powerful approach for HCP analysis. Untargeted acquisition strategies such as data-dependent acquisition (DDA) and data-independent acquisition (DIA) are commonly employed to profile HCP populations and to assess their clearance along purification processes [15,16,17,18,19]. While these approaches provide broad proteome coverage, they often require extensive sample preparation, long analysis times, and complex data processing, which may limit their applicability for routine or high-throughput studies.

Targeted proteomics based on selected or multiple reaction monitoring (SRM/MRM) has consequently gained increasing interest for the quantification of predefined HCP panels [20,21]. When combined with stable isotope-labeled internal standards, MRM offers high specificity, sensitivity, and quantitative robustness over a wide dynamic range. Moreover, MRM methods can be applied on low-resolution instruments such as triple quadrupoles (TQ), which are easily implementable in any laboratory and recognized for being particularly robust and reproducible [22,23,24]. These features make targeted LC–MS workflows particularly well-suited for the monitoring of known high-risk or process-relevant HCPs. However, conventional targeted methods typically rely on retention time (RT) scheduling to multiplex large numbers of transitions, rendering them sensitive to chromatographic variability.

In complex bioprocess-related matrices, peptide RTs may vary substantially due to differences in sample composition, peptide load, or chromatographic conditions [25]. Such variations can lead to truncated chromatographic peaks or complete signal loss in RT–scheduled MRM assays, especially when highly multiplexed methods are transferred across heterogeneous matrices. This dependence on RT stability represents a major limitation for the robustness and transferability of targeted proteomics workflows.

To overcome these limitations, event-driven acquisition strategies have been proposed as an alternative to fixed RT scheduling.

Escher et al. [26] described a method for RT calibration called iRT. It consists of adding standard peptides to the sample to predict the RT of endogenous peptides. This approach needs a first injection to determine the RT of the standard peptides, to then predict the RTs. The predicted RTs are then used to create the targeted method. A disadvantage is that 2 injections are needed for each matrix.

Gallien et al. [27] reported a method where the instrument operates in two alternating modes. A watch mode focuses on the fast, real-time detection of internal standards. The detection of the standard triggers the quantitation mode of the corresponding endogenous peptides during their elution time. One limitation of this approach is that acquisition of the target analyte only starts after the triggering event, leading to partial loss of the chromatographic peak.

Remes et al. [28] proposed a real-time alignment strategy that relies on a prior reference run to refine scheduling windows. This approach uses signals from background matrix components as alignment markers, enabling dynamic correction of retention times during acquisition. It is particularly well-suited for cohorts composed of similar sample types, such as plasma samples. However, this strategy requires an additional injection and the availability of a reference or control sample with a matrix composition identical to that of all subsequent samples, which constitutes a key limitation.

Recently, we introduced scout-triggered MRM (st-MRM), which relies on the detection of predefined scout peptides to dynamically activate groups of transitions, enabling targeted monitoring independently of absolute RTs [29,30,31,32,33]. This concept allows large, multiplexed assays to be performed in a single injection while maintaining robustness against chromatographic shifts.

In the present study, we apply st-MRM to the targeted quantification of host cell proteins as a representative and analytically challenging use case. A highly multiplexed assay targeting 97 CHO-derived HCPs (240 peptides, 720 transitions) was developed and evaluated across multiple bioprocess-related matrices. We demonstrate that conventional RT–scheduled MRM methods lack transferability between such matrices, whereas st-MRM enables consistent peptide detection and absolute quantification without method re-optimization. These results highlight the potential of event-triggered targeted proteomics as a robust and pragmatic solution for high-multiplex protein quantification in complex biological matrices.

## 2. Materials and Methods

### 2.1. Reagents and Chemicals

Water (H_2_O), acetonitrile (ACN), and methanol (MeOH) were obtained from Thermo-Fisher Scientific (Waltham, MA, USA) (LC-MS grade). Urea, ammonium bicarbonate, formic acid (FA),Trifluoroacetic acid (TFA), dithiothreitol (DTT), and iodoacetamide (IAA) were obtained from Sigma-Aldrich (St. Louis, MO, USA). Trypsin was obtained from Roche (Mannheim, Germany).

### 2.2. Sample Preparation

#### 2.2.1. Recombinant Therapeutic Protein Samples from CHO Cell Cultures

Recombinant proteins from CHO cell cultures were harvested at various stages of purification (DSP) by various biomanufacturing industries (from cell culture harvest to purified pharmaceutical substances). Prior to experiments, samples were stored at −20 °C according to supplier recommendations.

#### 2.2.2. Quantification Standards

The standard quantification peptides used in this study are heavy peptides with the same sequence as the endogenous peptides, containing a C-terminal lysine or arginine [^15^N and ^13^C]. The peptides were synthesized by Thermo-Fisher Scientific and SB-PEPTIDE (Saint Egrève, France) (both, purity > 50%; exact concentration was evaluated as described in Section 2.3) and stored at −20 °C until use.

The peptides were then adsorbed from water-soluble polymer beads using the patented READYBEADS™ technology from ANAQUANT (Lyon, France).

#### 2.2.3. Scheduled MRM

A total mass of 500 µg of total protein from the samples was denatured in 8 M urea, reduced with 20 mM DTT for 40 min at 60 °C, and alkylated with 50 mM IAA for 40 min in the dark at room temperature. An overnight digestion at 37 °C with trypsin at a 1:20 enzyme to total protein ratio (*w*/*w*) was performed. The heavy isotope-labeled peptide standard was added at the end of the process. The sample was then acidified with FA (final 0.5% *v*/*v*) to stop the digestion reaction, and the peptide mixture was desalinated on a C18 3CC cartridge according to the supplier’s protocol (conditioning the cartridge, sample loading, washing with acidified water, and elution with a high percentage of organic solvent). The SPE eluate solvent was evaporated to dryness using a miVac concentrator from Genevac (Warminster, PA, USA) operated at 35 °C for 3.5 h. The samples were stored dry at −20 °C. The peptides were then rehydrated with the injection solvent and Scout peptides on the day of analysis.

#### 2.2.4. st-MRM

Same procedure as described in scheduled MRM.

#### 2.2.5. DDA

A total mass of 150 µg of total protein was prepared using the same digestion protocol described above.

The sample was then fractionated at high pH in NH_4_OH to pH = 10 on a 15 cm × 2.1 × 3.5 µm Waters BEH300 column at 300 µL/min with post-digestion sample injection with online SPE (C18) Waters (Milford, MA, USA). An Agilent 1260 HPLC system (Santa Clara, CA, USA) with a fractionator was used to collect 24 fractions of 300 µL, which were then combined into 8 final fractions. The gradient was from 2 to 35% ACN. These 8 fractions were injected for classic acid LC-MS/MS analysis on high resolution, and the identification/quantification of the 8 fractions was compiled to provide a single result.

### 2.3. LC-UV Analysis (Qualification of Heavy Peptide Standards)

Quality control analyses of the heavy peptides using a UV detector at 205 nm were performed on an Agilent 1260 system with an AERIS XBridge C18 100 × 2.1 mm, 3.5 µm column (Phenomenex, Le Pecq, France). The peptides were analyzed with a linear gradient from 5 to 35% ACN for 25 min at a flow rate of 0.3 mL/min. To ensure better peak resolution, solvent A was water containing 0.04% TFA, and solvent B was ACN containing 0.04% TFA.

The concentration of the analyte is determined using the absorbance A_λ_ at a wavelength λ according to Beer–Lambert’s law in Equation (1) [34]:

(1) $$A_{\lambda }=\varepsilon_{\lambda }cl$$

where ε_λ_ is the molar absorptivity at wavelength λ, c the concentration, and l the path length.

The molar extinction coefficient ε_205_ is calculated according to the formula in Equation (2) [35,36]:

(2) $$\varepsilon_{205}=\sum \left(\varepsilon_{i}n_{i}\right)+\varepsilon_{bb}(r-1)$$

where for each amino acid type i, ε_i_ is the molar absorptivity of that amino acid type, n_i_ is the number of times that amino acid type appears in the polypeptide sequence, ε_bb_ is the molar absorptivity for a single backbone peptide bond, and r is the number of residues in the polypeptide sequence.

Beer–Lambert’s law usually only applies when using cuvettes. Extrapolation is necessary in order to apply it to an LC chromatogram to obtain Equation (3) (Adaptation of Beer–Lambert’s law applied to peptide quantification by UV detection at 205 nm in liquid chromatography [37]):

(3) $$C=\frac{Height\times M\times \sqrt{2\pi }\times W_{0.5}\times Q}{\varepsilon_{205}\times l\times V_{inj}\times 2.35}$$

where M is the molecular mass of the peptide, W_0.5_ is the peak width at half height, Q is the analysis flow rate, ε_205_ is the molar extinction coefficient, l is the optical path length (always equal to 1 in this case), and V_inj_ is the injection volume.

The results of quantification can be found in Supplementary Table S1.

### 2.4. LC-MS Analysis

#### 2.4.1. Experimental Design and Replicates

Method development and performance evaluation were initially conducted using three representative samples, each analyzed in triplicate LC–MS injections to assess retention time stability, repeatability, and acquisition robustness of the st-MRM workflow. For the comparative and application-focused analyses presented in this manuscript, four additional independent samples were analyzed using a single LC–MS injection per acquisition mode and per sample.

This design was intentionally selected to reflect routine quality control (QC) practice, where analyses are typically performed using a single injection per sample and where methods are expected to perform reliably without repeated measurements. In this context, the st-MRM acquisition strategy provides an additional safeguard, as it enables adaptive transition triggering in the event of chromatographic retention time shifts, thereby mitigating common sources of analytical failure in single-injection workflows.

The relatively high protein amount required per analysis (500 µg × 2 per injection for both Scheduled and st-MRM methods, and 150 µg for the DDA method) further limited the feasibility of technical replicates for all samples.

#### 2.4.2. Scheduled MRM

##### Creation of the Targeted Assay

Based on the list of HCP reported as risky by the BioPhorum group [38] and internal knowledge from discovery proteomics, a panel of 97 proteins derived from CHO cells was established (full list can be found in Supplementary Table S2). Up to 3 reporter peptides per protein were chosen. The peptide selection was based on simple rules: (1) ionization efficiency, (2) peptide proteotypicity, (3) peptide sequence length (between 5 and 25 amino acids), and (4) exclusion of peptides containing methionine oxidation. In total, 120 peptides and their 120 heavy isotope-labeled standards (^13^C and ^15^N on lysine and arginine) were included in the final assay. The selected isotope-labeled peptides were analyzed by direct infusion on a hybrid triple quadrupole/linear ion trap QTRAP^®^ 6500 LC/MS/MS System (SCIEX, Concord, ON, Canada). Skyline 22.2 software (MacCoss Lab Software, Seattle, DC, USA) was used to generate a list of appropriate MRM transitions. From the initial set of candidate MRM transitions, 3 transitions per peptide were selected for the final assay. The declustering potential (DP) and collision energy (CE) were optimized for each peptide and transition. To ensure that the selected heavy MRM transitions did not interfere with endogenous MRM transitions, pools of synthesized heavy peptides were injected. The final method contains a total of 720 transitions.

##### LC-MS/MS Parameters

LC-MS/MS analysis was performed on an Exion LC system (SCIEX, Concord, ON, Canada) coupled to a hybrid triple quadrupole/linear ion trap QTRAP^®^ 6500 LC/MS/MS system (SCIEX, Concord, ON, Canada). The LC separation of the injected sample was performed on a Xbridge BEH 300 1 × 150 mm, 3.5 µm (Waters). Elution was carried out at a flow rate of 100 µL/min with H_2_O containing 0.1% FA as eluent A and ACN containing 0.1% FA as eluent B.

Since HCPs are often present at low concentrations compared to the drug substance, a relatively high protein load (400 µg per injection) was used to maximize HCP detectability. In this study, LC–MS/MS analyses were performed under conventional-flow conditions (100 µL/min), which offer higher robustness and reproducibility than nanoLC systems but inherently dilute analytes, thereby requiring higher injected amounts to achieve sufficient sensitivity.

Moreover, the method was designed to be applicable across a wide range of biotherapeutic matrices, from harvest cell culture fluids to highly purified drug substances, where residual HCPs may be present at the low ppm level. The injected protein amount reflects a deliberate compromise between analytical robustness, sensitivity, and broad applicability, rather than optimization for a single sample type.

Preliminary studies showed that most peptides were eluted between 12 and 22% B. Therefore, the chromatographic method used an isocratic gradient at 5% B for 2 min, followed by a gradient from 5 to 12% B in 6 min, from 12 to 22% B in 25 min, and finally from 22 to 35% B in 11 min. The column was washed with 98% B for 8 min and re-equilibrated to 5% B for 8 min. The total analysis time was 60 min. MS data were acquired in the positive mode with a nebulization voltage of 5500 V, nebulization gas pressure set to 60 psi, desolvation gas pressure to 70 psi, and a temperature of 550 °C. The collision cell entrance and exit potentials were set to 12 and 10 V, respectively. The curtain gas and collision gas pressures were set to 50 and 8 psi, respectively. The cycle time was optimized to 1800 ms to ensure at least 5 ms dwell time and at least 8 points per peak. Instrument control and data acquisition were performed using SCIEX OS™ 3.3 software. The detection window was set to 220 s, centered on the RT.

#### 2.4.3. st-MRM

##### Selection of Scout Peptides

The Scout peptides selected in this study are 4 stable exogenous peptides: FGQTPVQEGR, AGIPNNQVLGK, EGQLTPLIK, and SGIPDNAFQSFGR. These synthetic peptide sequences do not correspond to any known proteome and have already been described as “RT calibrators” by ANAQUANT. The Scout peptides were adsorbed from water-soluble polymer beads using the patented READYBEADS™ technology from ANAQUANT. The charged peptide amounts are evaluated by ANAQUANT to obtain an adequate MS signal in a complex matrix. The two best transitions without contamination were added to the st-MRM method (8 Scout transitions in addition to the 720 transitions of interest).

##### LC-MS/MS Parameters

Same as scheduled MRM parameters.

##### Creation of the st-MRM Method

Four Scout peptides were chosen to create 5 groups of 84, 156, 180, 132, and 168 transitions, respectively. The RT overlap parameter defines a tolerance around each transition so that any co-eluting transition with a Scout transition is acquired during two acquisition windows. This parameter is particularly valuable in situations where variations in selectivity are observed between different matrices. The RT overlap was set to 0.8 min.

#### 2.4.4. DDA

DDA analyses were performed on a Q-Exactive HF instrument (Thermo-Fisher Scientific, San Jose, CA, USA) coupled to an RSLC Ultimate 3000 nano system liquid chromatography system (Thermo-Fisher Scientific, San Jose, CA, USA). TOP20 parent ions were fragmented, with an isolation window of 2 *m*/*z*, NCE set at 27, and dynamic exclusion at 10 s. For peptide separation, a PepMap™ RSLC C18, 2 mm, 0.075 mm ID × 500 mm analytical column (Thermo-Fisher Scientific, Waltham, MA, USA) was used. Solvent A was H_2_O containing 0.1% FA, and solvent B was ACN containing 0.1% FA. Peptides were eluted with a gradient of 3% to 40% solvent B for 60 min at a flow rate of 300 nL/min. 250 ng of the sample was loaded onto the column.

### 2.5. Data Reprocessing

#### 2.5.1. Scheduled MRM

The SCIEX OS™ 3.3 software, using the Autopeak algorithm for peak integration, was used for data analysis. Integrations were checked, and some peaks were re-integrated if necessary.

#### 2.5.2. st-MRM

Same procedure as scheduled MRM.

#### 2.5.3. DDA

The UniProt database (SWISSPROT TrEMBL) was used to retrieve proteins expressed in *Cricetulus griseus* (CHO) cells. MS/MS spectra were assigned to the peptide sequence using a database search strategy with the X!Tandem search engine. Trypsin was defined as the enzyme, and two missed cleavages were allowed. Cysteine modification was fixed while methionine mono-oxidation was defined as a variable modification. A decoy strategy was used to ensure a false discovery rate (FDR) of less than 1%. The validation step was carried out using Proline v1.6.1 software [39].

## 3. Results and Discussion

### 3.1. Scheduled MRM

To meet pharmacopeial expectations, the objective of this study was to develop an absolute quantification method for host cell proteins (HCPs) using targeted mass spectrometry in multiple reaction monitoring (MRM) mode. This method involved monitoring 120 HCP-specific peptides with internal calibration using 120 stable isotope-labeled homologs. By default, one proteotypic peptide was judiciously chosen per protein. In addition, two to three peptides were chosen for high-risk proteins (for example, two peptides for the Lipoprotein lipase (LPL), Clusterin (CLU), and Procollagen-lysine 2-oxoglutarate 5-deoxygenase_1 (PLOD1), and three peptides for the Phospholipase B-like 2 (PLBL2)). The peptide selection was based on simple rules: (1) ionization efficiency, (2) peptide proteotypicity, (3) peptide sequence length (between 5 and 25 amino acids), and (4) exclusion of peptides containing methionine oxidation. Since three transitions per peptide were monitored, the method included a total of 720 transitions, necessitating an MRM acquisition mode where each peptide’s transitions were only tracked within an RTwindow (referred to as “Scheduled MRM” by SCIEX, “Timed MRM” by Thermo-Fisher, and “Dynamic MRM” by Agilent). The sum of the dwell time within an RT window defined the total cycle time, which needed to be compatible with recording at least eight data points to define a chromatographic peak.

In practice, transition monitoring windows are typically 5–10 times (2–5 min) larger than the chromatographic peak width to account for RT drifts. To determine the RT of each target peptide (heavy and corresponding light peptides have the same RT), the transition list was divided into four separate MRM methods. All peptide RTs were established from the heavy RTs (spiked) in the Drug Substance 1 matrix, a single “Scheduled MRM” method was created with a dwell time of 5 ms, an interscan time of 5 ms, and a maximum cycle time of 1800 ms, ensuring approximately 15–16 data points per peak (peak width at baseline ~30 s). Under this configuration, Equation (4) resulted in a detection window of 220 s (3.7 min), more than seven times the peak width. Such a broad detection window should have allowed unbiased peptide detection, with a tolerance for RT shifts of approximately ±90 s around the expected RT (Relationship between dwell time, total cycle time, number of transitions per window, and inter-cycle time to optimize detection window).

(4) $$Dwell\;time=\frac{Cycle\;time}{Number\;of\;transitions\;per\;windows}-inter-cycle\;time$$

**Figure 1:**
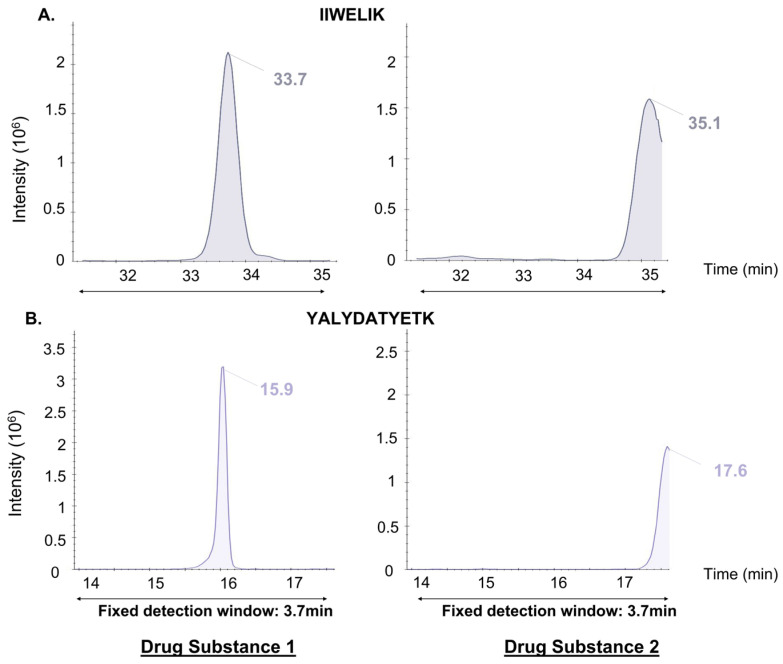
RT shifts of the same peptides in two different drug substances using the Scheduled MRM. Ion chromatograms (sum of three transitions) showing examples of RT shifts for two targeted peptides ((**A**) IIWELIK and (**B**) YALYDATYETK) monitored by Scheduled-MRM in purified drug substances matrices 1 and 2. The peptides chromatograms on the left are obtained from their adapted method. The same method was applied to another purified drug substance matrix, and the results for the corresponding peptides are presented on the right. When the matrix changes, an RT shift occurs (1.4 min for (**A**) and 1.7 min for (**B**)) and induces truncation of the chromatographic peaks.

**Figure 2:**
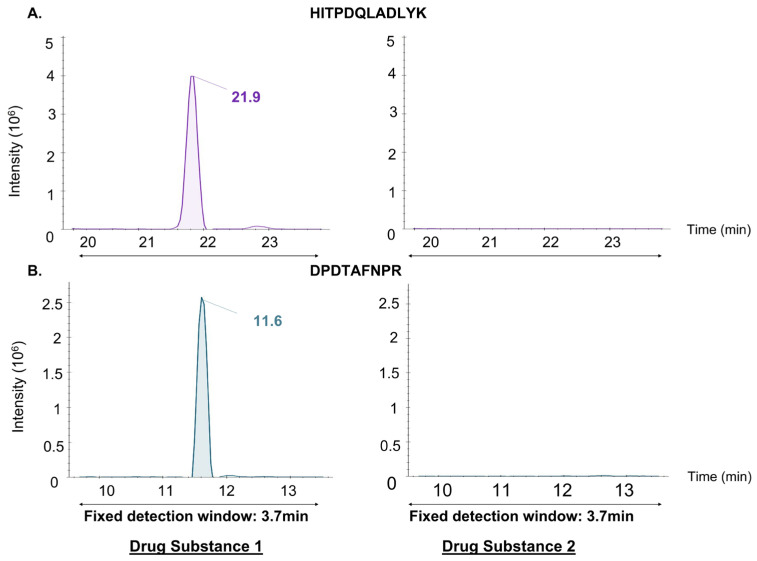
RT shifts of the same peptides in two different drug substances using the Scheduled MRM. Ion chromatograms (sum of three transitions) show examples of RT shifts for two targeted peptides ((**A**) HITPDQLADLYK and (**B**) DPDTAFNPR) monitored by Scheduled-MRM in purified drug substance matrices. The peptide chromatograms on the left were obtained from their adapted method. The same method was applied to another purified drug substance matrix, and the results for the corresponding peptides are presented on the right. When the matrix changes, RT shifts occur, and peptides are eluted out of the fixed detection window.

This “Scheduled MRM” method was then evaluated by analyzing four extracts from different stages of therapeutic protein production derived from CHO cell cultures. Figure 1 and Figure 2 demonstrate that extrapolating an optimized method from one matrix (e.g., Drug Substance from one manufacturer) to another matrix with a different composition (e.g., Drug Substance from another manufacturer) led to biased detection of certain target peptides. Chromatographic peaks of some peptides appeared truncated (IIWELIK; RT shift of 1.4 min, YALYDATYETK; RT shift of 1.7 min), while others were not detected due to elution outside the detection window (HITPDQLADLYK, DPDTAFNPR).

Among the 120 heavy peptides spiked into Drug Substance 2 and monitored using the “Scheduled MRM” method developed from the Drug Substance 1 matrix, 18 peptides (15%) exhibited truncated chromatographic peaks, while 48 peptides (40%) were not detected as they eluted outside their detection window. In total, only 54 peptides (45%) were correctly eluted within their designated windows. These significant RT shifts in different extracts were primarily due to variations in the relative concentrations of different peptides. Additionally, the need to inject a high amount of solute (400 µg) to achieve a dynamic range spanning six orders of magnitude led to column overloading [25].

The analysis of 120 heavy peptides, selected as reporters for 97 HCPs, using “Scheduled MRM,” proved problematic across different matrices, necessitating systematic method adjustments for each sample.

**Figure 3:**
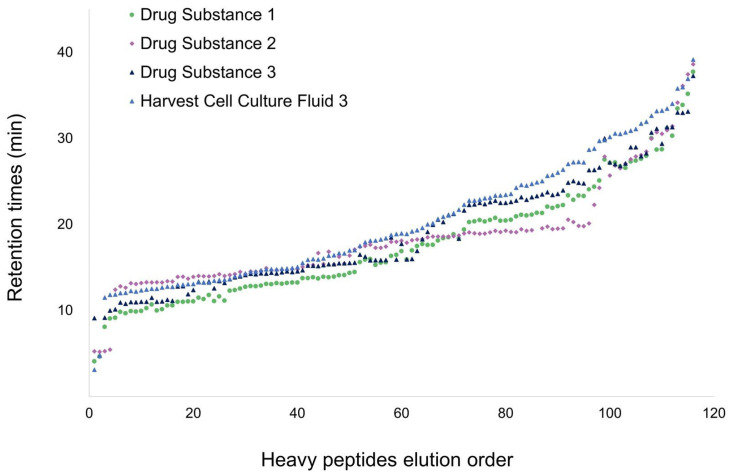
RTs and elution order of 120 heavy peptide standards, used to quantify 97 HCP, in the four different samples. Drug Substances 1, 2, and 3 are different purified products. Harvest Cell Culture Fluid 3 is the same product as Drug Substance 3, collected before the purification steps.

To assess the extent of RT variations, heavy peptide RTs were collected from four different matrices: three purified therapeutic proteins (Drug Substances) and one harvest cell culture fluid from three different manufacturers. Significant RT shifts were observed between matrices, as illustrated in Figure 3, with RT deviations of up to 8 min across all samples and up to 6 min between two purified Drug Substances. Such RT shifts make it impossible to use “Scheduled MRM” as a universal method for quantifying HCPs in recombinant proteins derived from CHO cell cultures.

This highlights the difficulty of implementing and sharing these assays due to their dependency on RT, which is known to vary between chromatographic systems and sample matrices. This first phase of development shows that it is not feasible to parameterize a single “Scheduled MRM” assay to screen HCP reporter peptides in different therapeutic protein production matrices.

### 3.2. st-MRM

In the context of unpredictable RT shifts, the st-MRM acquisition method proves highly effective [29,30,31,32,33]. st-MRM is available in SCIEX OS acquisition software, making it easy to build highly multiplexed methods. Essentially, it involves a group of transitions triggered successively by scout compounds strategically placed throughout the chromatogram and activated once detected above a predefined threshold.

This approach ensures reliable and robust detection despite RT drifts. These scouts can be endogenous or spiked molecules. If a molecule co-elutes with a detection compound, the transitions for that molecule are tracked within the two contiguous transition groups to define the chromatographic peak perfectly.

In our case study, all transitions tracking both endogenous light peptides and standard heavy peptides were positioned in five distinct groups, four of which were triggered by signals from eight transitions of four exogenous spiked peptides used as scouts. These scouts were carefully selected based on their relative RT, ensuring that each group monitored no more than two competing transitions per peptide.

**Figure 4:**
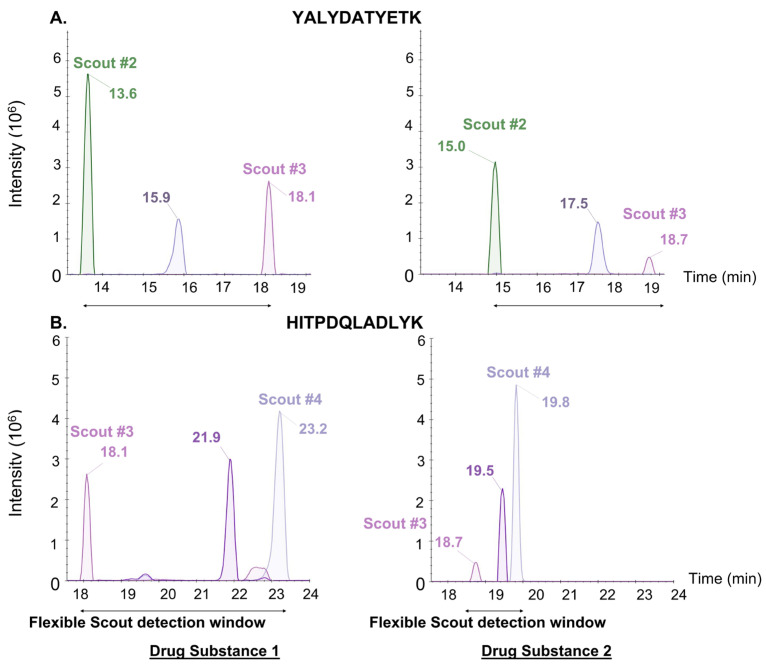
RT shifts of the same peptides in two different drug substances using the st-MRM. Ion chromatograms showing examples of RT shifts for two targeted peptides ((**A**) YALYDATYETK) and ((**B**) HITPDQLADLYK) monitored by the st-MRM method. In case of any RT shift in st-MRM methods, Scout peptides automatically realign the detection window.

A first st-MRM method was developed using the relative RT of the heavy peptides spiked in Drug Substance 1. The detection recovery was then evaluated in three other matrices (Drug Substance 2, Drug Substance 3, and Harvest Cell Culture Fluid 3). Despite RT variations, 100% of the heavy peptides were detected in each matrix, demonstrating the extreme robustness of the st-MRM method against chromatographic disruptions. Figure 4 shows how Scout peptides automatically realign the detection window between Drug Substance 1 and Drug Substance 2. This also demonstrates that selectivity is preserved between the two different drug substances in this case. In particular, the elution order of Scout #2, YALYDATYETK, Scout #3, HITPDQLADLYK, and Scout #4 is conserved.

#### 3.2.1. Detection Performance of HCPs

Samples analyzed using st-MRM were also analyzed with DDA, with prior high-pH reversed-phase fractionation, and identification and quantification based on the TOP3 strategy [40], to compare detection of the 97 HCPs (via the detection of the reporter peptides) in st-MRM versus detection of proteins from this list in DDA.

**Figure 5:**
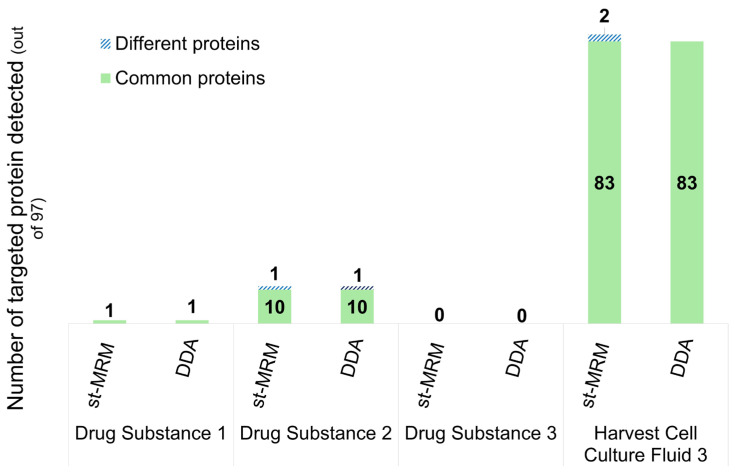
Comparison of the number of targeted HCPs detected across different purification steps and samples (Drug Substance 1, Drug Substance 2, Drug Substance 3, and Harvest Cell Culture Fluid 3), using both Scout-triggered MRM (targeted) and fractionated DDA (untargeted) approaches. In Drug Substance 1, the st-MRM and DDA methods detected the same protein. In Drug Substance 2, st-MRM and DDA detected 10 common proteins and detected one more protein that was not detected by the other. In Drug Substance 3, neither method detected any proteins. In Harvest Cell Culture Fluid 3, both methods detected 83 common proteins, and st-MRM detected two proteins that were not detected with DDA. Light blue indicates proteins uniquely detected by st-MRM, whereas dark blue indicates proteins uniquely detected by DDA.

Figure 5 presents the number of proteins detected from the list of 97 in st-MRM versus DDA, across four different samples: three purified therapeutic proteins (drug substances) and one harvest cell culture fluid from three manufacturers (the same four samples used in Figure 3).

First, it is noteworthy that the st-MRM method has good specificity. In the Drug Substance 3 sample, where the DDA fractionation approach did not identify any proteins from the list, no proteins were detected in st-MRM either. The same applied to Drug Substance 1, with only one protein identified in DDA and the same protein detected in st-MRM.

**Figure 6:**
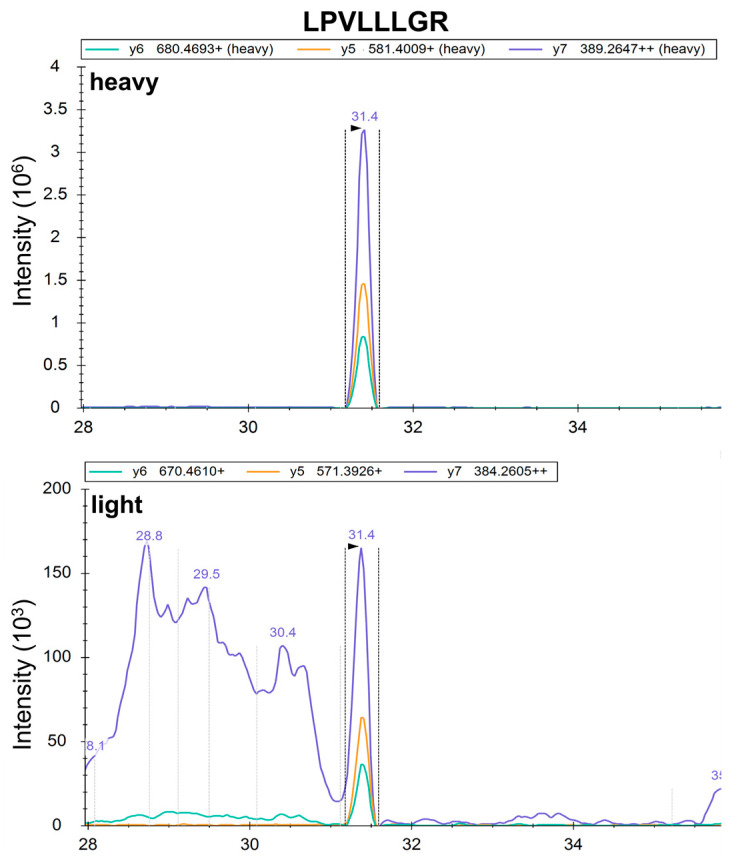
Confirmation of peptide detection in st-MRM. Ion chromatograms of the heavy and light LPVLLLGR peptides showing the same RT and the same transition ratio, confirming the correct detection of the protein associated with the peptide.

For Drug Substance 2, 10 common proteins were detected by both methods. However, one peptidase protein detected in st-MRM was not detected in DDA. st-MRM chromatograms for the peptide used as a reporter for this protein are shown in Figure 6. The characteristics of the heavy standard peptide unambiguously confirm the presence of the light peptide and hence the protein (same RT, same transition ratio), demonstrating the sensitivity of st-MRM, compared with DDA on a high-resolution instrument.

On the other hand, a dehydrogenase protein was detected in DDA but not in st-MRM. This protein was identified in DDA by two specific peptides and quantified below the lower limit of quantification (LLOQ), thus at the detection limit. The st-MRM method of fractionation was less sensitive in this case and failed to detect the protein. However, since it was below the LLOQ, this would not affect the overall quality control limit, which remains below 100 ppm.

For the Harvest Cell Culture Fluid 3 sample, 83 common proteins were detected by both methods. Two additional proteins were detected in st-MRM but not in DDA. Two peptides per protein were chosen in this case, confirming the protein detection unequivocally.

In summary, the st-MRM method showed 99% overlap with the DDA method, with the advantage of requiring much simpler and faster sample preparation, without fractionation. These results demonstrate the specificity, sensitivity, and relevance of targeted approaches for the reliable detection of proteins.

#### 3.2.2. Quantification Performance of HCPs

The detected proteins were then quantified, absolutely in st-MRM and relatively in DDA, respectively (quantification method described by Trauchessec et al. [41] for DDA). Briefly, this method (DDA) uses a standard with 54 peptides representing 18 proteins as an internal calibration curve. Notably, this relative quantification is limited by the calibration curve (1–500 fmol injected), while the absolute quantification in st-MRM is only limited by the instrument’s sensitivity for low-concentration proteins.

**Figure 7:**
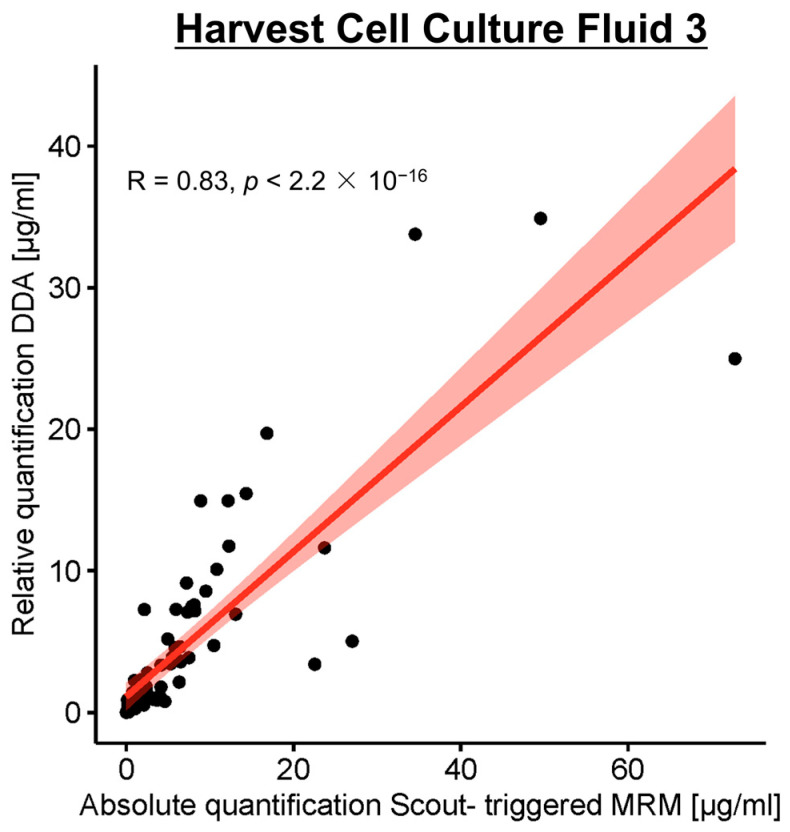
Pearson correlation coefficient scatter plot between HCP quantity measured using Scout-triggered MRM and DDA in the Harvest Cell Culture Fluid 3 sample. The Pearson coefficient shows a correlation characterized by R = 0.83, *p* < 2.2 × 10^−16^.

Qualifications were compared with data from the Harvest Cell Culture Fluid 3 sample in µg/mL (83 common proteins). A correlation plot is shown in Figure 7. The Pearson coefficient calculated shows a strong positive correlation (R = 0.83, *p* < 2.2 × 10^−16^) between the two quantification methods. However, the absolute quantification by st-MRM allows for the correction and clarification of some values, particularly those outside the DDA calibration curve.

**Figure 8:**
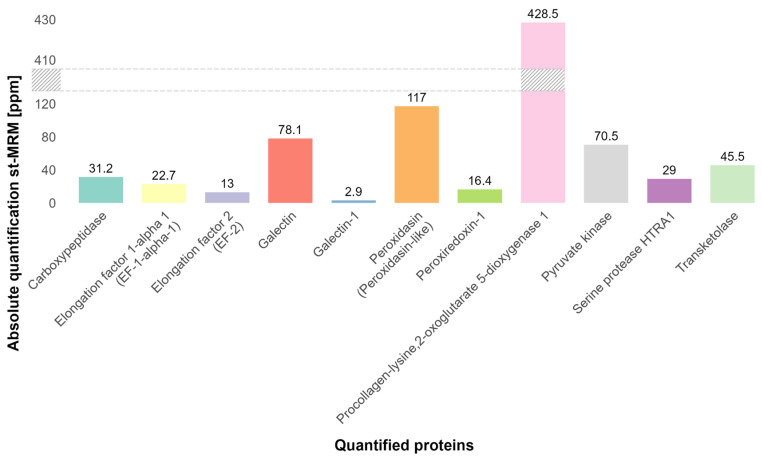
Absolute quantification of targeted proteins in Drug Substance 2 by st-MRM (ppm). A total of 11 proteins (Carboxypeptidase, Elongation factor 1-alpha 1 (EF-1-alpha-1), Elongation factor 2 (EF-2), Galectin, Galectin-1, Peroxidasin (Peroxidasin-like), Peroxiredoxin-1, Procollagen-lysine,2-oxoglutarate 5-dioxygenase 1, Pyruvate kinase, Serine protease HTRA1, Transketolasewere) were quantified from 2.9 to 428.5 ppm. The grayed region represents a break in the scale.

To evaluate the dynamic range achieved with the st-MRM method, proteins from Drug Substance 2, purified to 99%, were quantified absolutely in ppm (Figure 8, the grayed region represents a break in the scale). In this sample, the lowest-abundance HCP was quantified at 2.9 ppm, corresponding to an injected amount of approximately 0.6 ng. Given a total injected protein load of 400 µg, of which ~99% corresponds to the drug substance, these results demonstrate that protein-level quantification was achieved over a dynamic range spanning six orders of magnitude between the lowest quantified HCP and the drug substance.

#### 3.2.3. Comparative Evaluation of Targeted and Untargeted Approaches

To provide a clear overview of the analytical performance, robustness, and practicality of different HCP quantification strategies, we compared st-MRM, RT-scheduled MRM, DDA, DIA, and conventional ELISA (Table 1). This comparison highlights that while DDA and DIA provide broad proteome coverage, they require extensive sample preparation or complex data analysis, and their quantitative accuracy is limited for low-abundance HCPs. Scheduled MRM can achieve absolute quantification but is highly sensitive to RT shifts across matrices. In contrast, st-MRM enables robust, absolute quantification of all target peptides in a single injection, with minimal sample preparation, high matrix transferability, and excellent quantitative performance. ELISA remains useful for total HCP assessment but cannot provide protein-specific information. Overall, st-MRM offers a practical and reliable solution for high-multiplex HCP monitoring in biopharmaceutical development.

## 4. Limitations and Perspectives

Despite the advantages demonstrated in this study, several limitations of the st-MRM workflow should be acknowledged. As a targeted proteomics approach, st-MRM relies on a predefined panel of proteins and peptides and, therefore, does not provide exhaustive coverage of the host cell proteome. Protein identification and quantification are performed at the peptide level and interpreted at the level of canonical protein entries; consequently, the approach does not aim at resolving the full diversity of host cell protein proteoforms arising from post-translational modifications, sequence variants, or proteolytic processing. It is not intended to replace untargeted discovery-based workflows, but rather to support the robust monitoring and quantification of predefined, process-relevant HCPs.

While event-triggered acquisition improves robustness against RT shifts, it does not inherently increase analytical sensitivity. Compared with deeply fractionated or extended-gradient DIA workflows, the sensitivity of the approach remains constrained by the single-injection LC–MS setup used here, which may limit the detection of very low-abundance HCPs in highly purified samples.

Despite these limitations, st-MRM offers several perspectives for the future development of robust targeted proteomics workflows. The ability to decouple transition acquisition from fixed RT scheduling represents a significant conceptual advance for highly multiplexed assays applied across heterogeneous biological matrices. This feature is particularly relevant for bioprocess monitoring, where sample composition may vary substantially between process steps, batches, or purification conditions.

Future work could focus on extending the st-MRM concept to larger and more diverse protein panels, including HCPs from different expression hosts or additional classes of process-related impurities. In addition, untargeted proteomics approaches such as DDA or DIA could be leveraged upstream to identify critical HCP proteoforms, enabling the selection of representative peptides for subsequent targeted monitoring by st-MRM. The integration of automated scout peptide selection and data-driven assay optimization could further improve method robustness and facilitate assay transfer between laboratories.

From an application standpoint, st-MRM should be viewed as a complementary tool to existing analytical methods, including ELISA and untargeted LC–MS workflows, rather than as a direct replacement. In this context, st-MRM provides protein-specific quantitative information that can support risk-based decision-making and deepen process understanding.

Overall, the results presented in this study demonstrate that event-triggered targeted proteomics represents a promising and pragmatic solution for high-multiplex protein quantification in complex matrices. By addressing a key limitation of conventional RT–scheduled MRM, st-MRM acquisition strategies may contribute to improving the robustness, transferability, and applicability of targeted LC–MS workflows in bioprocess and proteomics applications.

## 5. Conclusions

The monitoring of HCPs throughout bioprocess development and purification remains a critical analytical challenge for the biopharmaceutical industry. In this study, we developed a targeted LC–MS/MS workflow for the detection and absolute quantification of predefined HCPs in biotherapeutic products, with the objective of providing protein-specific information complementary to routinely used immunoassay-based measurements.

A highly multiplexed assay targeting 97 CHO-derived HCPs was established using st-MRM, enabling the simultaneous monitoring of 240 peptides and 720 transitions within a single injection. Compared with conventional RT–scheduled MRM approaches, the event-triggered acquisition strategy improved robustness against RT variability and reduced the risk of data loss when analyzing heterogeneous bioprocess-related matrices. Under the experimental conditions investigated, the method enabled absolute quantification of HCPs over a broad dynamic range, with detection limits reaching the low parts-per-million range.

In addition to robustness, st-MRM substantially reduced overall analysis time and sample consumption. While highly multiplexed conventional MRM assays require multiple injections and method iterations to accommodate large numbers of transitions and compensate for RT shifts, the st-MRM workflow allowed the complete analysis of a sample in a single 60-min LC–MS run using reduced amounts of starting material and internal standards. This streamlined acquisition and data processing workflow highlights the practical advantages of event-triggered targeted proteomics for high-multiplex protein quantification.

Overall, this work demonstrates that st-MRM represents a robust and pragmatic targeted proteomics strategy for the quantitative monitoring of predefined HCP panels in complex bioprocess matrices. By alleviating key limitations associated with RT–scheduled acquisition, event-triggered approaches may facilitate the broader application of targeted LC–MS workflows in bioprocess development and analytical proteomics.

## Figures and Tables

**Table 1:** Comparative overview of HCP quantification methods.

Feature	st-MRM	Scheduled MRM	DDA	DIA	ELISA
Peptides/proteins quantified	97 proteins, robust across matrices	97 proteins, 40–55% peptides detected in other matrices	Broad coverage, many proteins	Broad coverage, many proteins, but complex data processing	Total HCP only, no protein-specific info
Matrix compatibility	Multiple Drug Substances and Harvest Cell Culture Fluid, 100% peptides detected	Requires re-optimization for each matrix	Limited by sample prep/fractionation	Can profile multiple matrices, but is sensitive to sample complexity	High, but not protein-specific
Number of injections	1	1–4 depending on RT shifts	8 fractions × 1 injection = 8 injections	1–2 injections, depending on setup	1 (plate-based)
Analysis time per sample	60 min	60–240 min depending on re-injections	8 × 60 min = 8 h	60–120 min	2–4 h including incubation
Requirement for fractionation	None	None	Required for deep coverage	Optional, usually none	Not applicable
Robustness to RT shifts	High, 100% peptides detected	Low	Not applicable	Not applicable	Not applicable
Quantitative type	Absolute (SIL peptides)	Absolute if detected	Relative, less precise	Relative, less precise	Relative/total HCP
Ease of method transfer	High	Low	Medium	Medium	High

## Data Availability

All the raw data have been deposited to the ProteomeXchange Consortium via the PRIDE [42] partner repository (https://www.ebi.ac.uk/pride/, accessed on 10 February 2026) with the dataset identifier PXD066465.

## Supplementary Materials

The following supporting information can be downloaded at: https://www.mdpi.com/article/10.3390/proteomes14010009/s1. Table S1: Qualification of the heavy standard peptides by LC-UV; Table S2: HCPs included in the assay.

## Abbreviations

The following abbreviations are used in this manuscript:

HCPs	Host Cell Proteins
mAbs	Monoclonal Antibodies
CHO	Chinese Hamster Ovary
LPL	Lipoprotein Lipase
PS-20	Polysorbate 20
PS-80	Polysorbate 80
FDA	Food and Drug Administration
EMA	European Medicines Agency
ELISA	Enzyme-Linked Immunosorbent Assay
LC-MS/MS	Liquid chromatography coupled to tandem mass spectrometry
DDA	Data-Dependent Acquisition
DIA	Data-Independent Acquisition
SRMS/MRM	Selected/Multiple Reaction Monitoring
TQ	Triple Quadrupoles
st-MRM	Scout-triggered MRM
RT	Retention Time
